# The time course of holistic processing is similar for face and non-face Gestalt stimuli

**DOI:** 10.3758/s13414-021-02415-w

**Published:** 2022-04-22

**Authors:** Kim M. Curby, Lina Teichmann

**Affiliations:** 1 School of Psychological Sciences, Macquarie University, Sydney, NSW 2109 Australia; 2 Centre for Elite Performance, Expertise, & Training, Macquarie University, Sydney, Australia

**Keywords:** holistic perception, time course, perceptual grouping, Gestalt perception, face perception

## Abstract

There is evidence that holistic processing of faces and other stimuli rich in Gestalt perceptual grouping cues recruit overlapping mechanisms at early processing stages, but not at later stages where faces and objects of expertise likely overlap. This has led to suggestions of dual pathways supporting holistic processing; an early stimulus-based pathway (supporting processing of stimuli rich in perceptual grouping cues) and an experience-based pathway (supporting processing of object of expertise), with both pathways supporting face processing. Holistic processing markers are present when upright faces are presented for as little as 50-ms. If the overlap between holistic processing of faces and stimuli rich in grouping cues occurs early in processing, markers of holistic processing for these Gestalt stimuli should be present as early as those for faces. In Experiment 1, we investigate the time-course of the emergence of holistic processing markers for face and non-face Gestalt stimuli. The emergence of these markers for faces and the Gestalt stimuli was strikingly similar; both emerged with masked presentations as little as 50-ms. In Experiment 2, where the stimulus presentation was not masked, thus the presentation duration, but not the post-presentation perceptual processing, was constrained, patterns of holistic processing for these stimuli still did not diverge. These findings are consistent with an early, and possibly extended, temporal locus for the overlap in the holistic processing of faces and non-face stimuli rich in grouping cues.

The degree to which the processing of face and non-face stimuli share overlapping features, whether that be at a neural and/or a cognitive mechanistic level, has been the topic of a lively ongoing debate in the literature (e.g., Kanwisher, 2017; McGugin et al., 2017). The answers have potential implications for our understanding of the cognitive and neural architecture of the core systems underlying visual information processing. One key feature of face processing that has been at the centre of this debate is holistic processing. Findings of similar markers of holistic processing for face and non-face object of expertise has led to the suggestion that this feature of face processing may be a result of our typically extensive experience individuating these stimuli. However, more recently, surprising evidence of holistic processing has emerged for non-face stimuli that are strong in perceptual grouping (Gestalt) cues (Zhao et al., 2016). Notably, unlike that for objects of expertise, the participants in these studies had no prior experience with these stimuli. The degree to which holistic processing of these stimuli reflects the use of the same mechanisms as those for faces, or instead merely a similarity at the output, behavioural level, is unclear. Here we investigate a key aspect of holistic processing, namely its time course, for faces and these novel line pattern stimuli rich in Gestalt grouping cues. Investigating the time course of holistic processing for these stimuli has the potential to provide insight into whether the holistic processing of faces and these novel Gestalt stimuli draw on common or distinct mechanisms.

Gestalt grouping cues allow us to perceptually organise features within our visual world into perceptual wholes (see Wagemans, Elder, et al., 2012a; Wagemans, Feldman, et al., 2012bfor a review). In general, these cues serve to inform the visual system that different perceptual features or items belong together either as related parts of a bigger pattern, or, in some cases, as parts of the same singular perceptual unit or object. Given that holistic processing is often considered the processing of stimuli as perceptual units, rather than as collections of features, holistic perception and Gestalt grouping appear to, at least at an intuitive level, share overlapping characteristics. The findings of more recent research provide support for an overlap that extends beyond mere intuition, potentially to the very mechanisms that support these two phenomena (Curby & Moerel, 2019).

Consistent with the proposal that holistic processing of face and non-face stimuli rich in Gestalt grouping cues relies on overlapping mechanisms, a recent study from our lab provided evidence of interference between the concurrent processing of these stimuli (Curby & Moerel, 2019). Faces were processed less holistically when novel stimuli rich in Gestalt grouping cues where overlaid on them, compared to when versions of these same stimuli, but with their Gestalt cues disrupted, were overlaid. Gestalt cues in these novel line drawings were disrupted by misaligning the top and bottom parts of the stimuli. Consistent with the reduction in holistic processing arising from competition for the holistic processing-related resources between the face and line stimuli, this interference was symmetrical with intact face stimuli, but not misaligned face stimuli, reducing holistic processing of the line stimuli. These findings provide support for the recruitment of overlapping resources, specific to holistic processing, by face stimuli and non-face stimuli rich in Gestalt cues.

If the holistic processing of face and non-face Gestalt stimuli overlap at early processing stages, markers of holistic processing for these stimuli should emerge similarly early for both types of stimuli. There are a number of measures or markers of holistic processing that have been explored extensively in the face recognition literature. One of the most robust is evident in the *composite task*, which reveals people’s obligation to process intact faces (and other objects of expertise; e.g., Curby & Gauthier, 2014) as wholes: when making a judgment about one half of the face, they experience interference from the other, task-irrelevant half (Young et al., 1987). Building on this original observation, the degree to which manipulations of the task-irrelevant part impact judgments about the task-relevant part provides an index of holistic perception (Richler & Gauthier, 2014). Part-matching judgments about composite faces are influenced by the compatibility of the task-irrelevant face parts (i.e., whether it would require the same [congruent] or a different [incongruent] response as that for the task-relevant part; *congruency effect*). This effect has also been shown to be attenuated when the top and bottoms parts are misaligned, disrupting the prototypical configuration of the face.

The composite face task has been used in studies directly probing the time-course of holistic processing of upright and inverted faces (Richler et al., 2009; Richler et al., 2011). Markers of holistic processing were found for upright faces when they were presented for as little as 50 ms (Richler et al., 2009; Richler et al., 2011), while holistic processing of inverted faces was delayed, relative to that for upright faces (Richler et al., 2011). The faster holistic processing for upright, compared to inverted, faces has been suggested to be a result of increased processing efficiency resulting from our extensive experience with upright faces. However, the emergence of markers of holistic processing under very rapid stimulus presentations conditions could also be interpreted as consistent with a role of early, fast-paced, perceptually-driven mechanisms in supporting holistic processing of faces.

Markers of perceptual grouping have been documented to occur on a similar timescale as the congruency effect for faces (e.g., Hadad & Kimchi, 2008; Kimchi & Hadad, 2002). For example, when grouping cues such as collinearity and/or closure are present, the configuration of disconnected line segments is rapidly made available and can prime responses to this configuration after as little as a 40 ms exposure (Hadad & Kimchi, 2008; Kimchi, 2000). Notably, this time course is equivalently rapid as that when the configurations or objects are defined by connected line segments (i.e., uniform connectedness). Therefore, we might expect that the perceptual grouping cues at work when perceiving Zhao et al. (2016) novel line pattern stimuli will operate on a similarly rapid timescale.

However, there is evidence that not all grouping cues can operate over the same time course, with some requiring additional time. For example, the time required for grouping of spatially disconnected elements was shown to differ depending on which grouping cue was operating: proximity-based grouping emerged after approximately 88 ms, while alignment-based grouping emerged later at approximately 119 ms (Kurylo, 1997). Notably, while markers of holistic processing of faces emerged with as little as a 50 ms presentation, such markers grew more robust up until about 150-200 ms (Richler et al., 2009). Thus, the variety of perceptual grouping cues present within complex stimuli like faces, and their different time courses, provides a potential account of both the early onset and the increase in indices of holistic processing over the initial period of processing.

Here we assess whether markers of holistic processing are present for face and non-face novel stimuli rich in Gestalt grouping cues under different temporal processing constraints. Richler et al. (2011) found that inverted faces required additional processing time before markers of holistic processing emerged. If slower holistic processing for inverted than upright faces is based on a lack of experience with inverted faces, holistic processing of *novel* line patterns should also be delayed. However, other findings suggest that the overlap between holistic processing of faces and stimuli rich in perceptual grouping cues occurs early in processing, potentially as early or earlier than the influence of experience on holistic processing (Curby et al., 2019; Curby & Moerel, 2019). If this is the case, markers of holistic processing of the novel line pattern stimuli should be present similarly early as those for upright faces, that is, after as little as a 50 ms masked presentation.

## Experiment 1

### Participants

Thirty-six undergraduate students (28 females, 8 males, mean age = 21.60, SD = 6.11) from Macquarie University participated for class credit. This sample size was chosen to match the sample size of the corresponding condition in Richler et al., 2011. All participants reported normal or corrected to normal vision and gave informed consent before participating.

### Stimuli

**Fig. 1:**
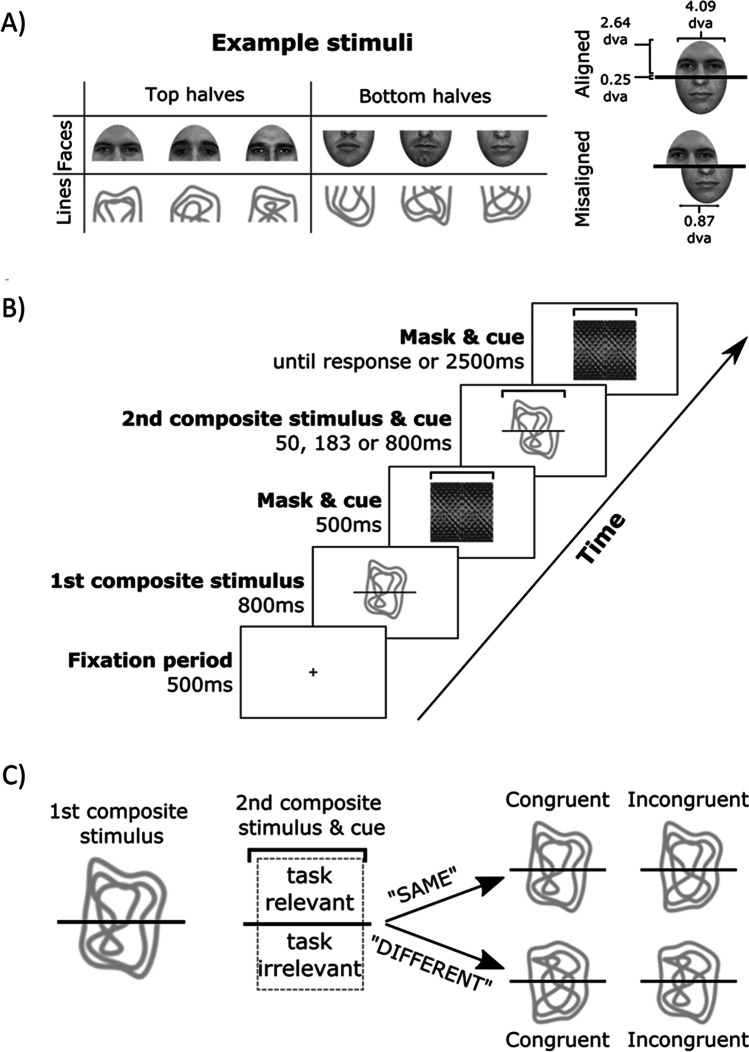
Example images and structure of the task trials. (A) shows three example stimuli top and bottom components for the face and line stimuli. In (B) we display the trial structure using an aligned, top cue, congruent, line trial. (C) shows an example of the congruency (i.e., “congruent” and “incongruent”) and response (i.e., “same” and “different”) mappings

The stimuli consisted of 20 greyscale front-view images of male faces wearing neutral expressions from Meissner et al. (2005) and 20 grey scale images of novel line patterns created to mimic those used in Zhao et al., 2016. The face images were cropped to remove the hair and ears. Both sets of stimuli were cut in half to obtain a top and bottom half (each part was ~ 5.2 x 4.1 dva [degree visual angle], Fig. 1a). Four additional face and line pattern stimuli were used in the practice trials.

### Design

A composite part-matching task with faces and novel line stimuli, with a 3 (duration; 50 ms, 183 ms, 800 ms) x 2 (congruency; congruent, incongruent) x 2 (alignment; aligned, misaligned) factorial design, was used. All variables were manipulated within-subjects. The dependent variables were sensitivity (d’) and response time (RT).

### Apparatus and Procedure

The stimuli were viewed on a 24-inch monitor, with a resolution of 1920 × 1080 pixels, at a distance of approximately 60cm. The experiment was programmed using Psychophysics Toolbox (PTB3) extensions of Matlab software (Brainard, 1997; Kleiner et al., 2007; Pelli, 1997).

The experiment consisted of 2 sessions completed on separate days, typically a week a part (mean interval = 6.78 days, *S.D*.=3.68). Participants performed the same part-matching task in both sessions. However, in one session the stimuli were composite faces and in the other they were composite novel line patterns. The order of the sessions was counterbalanced across participants.

The trials sequence was the same regardless of whether the composite stimuli were faces or line patterns (Fig. 1b). Each trial started with a central fixation cross (500 ms), followed by a composite (chimeric) stimulus comprising of the top and bottom parts of different faces or line patterns (800 ms). The composite stimuli were created by randomly combining the top of one face or line pattern with the bottom of another face or line pattern. A horizontal black line (width = 0.25 dva) was drawn in the middle in order to clearly separate the top and bottom halves. This was masked by a textured pattern (for 500 ms) that also contained either a bracket around the top or bottom part indicating which part was the task-relevant part. Following the mask, a second composite stimulus and the bracket cue were briefly presented (50 ms, 183 ms, or 800 ms). These presentation durations were selected to match those used in Experiment 2 of Richler et al., 2011 as the same three encoding durations were used in this study. In half the trials the top and bottom parts of this second stimulus were aligned and in the other half they were misaligned. This was followed by a mask with the bracket cue again until a response was made or 2500 ms had passed. Participants then indicated via a key press whether the cued half (top or bottom) of the second stimulus was the same or different to that of the first stimulus.

In 50% of trials the same/different relationship between the task-irrelevant (non-cued) stimulus parts in the two stimuli was *congruent* with the relationship between the task-relevant (cued) parts. In other words, in congruent trials, if the task-relevant parts differed between the two stimuli, thus rendering the correct response for the trial “different”, the task-irrelevant stimulus parts also differed. In the other trials, the same/different relationship between the task-irrelevant (non-cued) stimulus parts in the two stimuli was *incongruent* with the relationship between the task-relevant (cued) parts. For example, if the task-relevant parts differed between the two stimuli, thus again rendering the correct response for the trial “different”, the task-irrelevant stimulus parts were the same (Fig. 1c).

Before each experimental session, participants completed 48 practice trials. Participants completed eight blocks of 60 trials for a total of 480 trials in each of the two sessions. Participants were offered a break after every block**.** The order of trials was randomised. As in Richler et al. (2011), for each stimulus type (i.e., faces and line patterns) there were 10 trials for each combination of cued part (top/bottom), correct response (same/different), congruency (congruent/incongruent), alignment (aligned/misaligned) and presentation duration (50 ms/183 ms/800 ms). Sensitivity scores (d’) were calculated using the hit rate and false alarm rates for each condition and for each participant.

## Results & Discussion

Data from one participant was excluded due to poor performance (d’<0). Following procedures used in our previous studies (e.g., Curby et al., 2019), mean response times were calculated for correct trials only and a filter was applied to remove extreme RTs (i.e., RTs < 200 ms or >2000 ms). As expected, this filter resulted in minimal data loss (2.6 %). Following our preregistration, data from the face and line pattern conditions were analysed in separate 2 (congruency; congruent, incongruent) x 2 (alignment; aligned, misaligned) x 3 (duration; 50 ms, 183 ms, 800 ms) repeated measures ANOVAs.

### Face Stimuli

#### Sensitivity (d’)

**Fig. 2:**
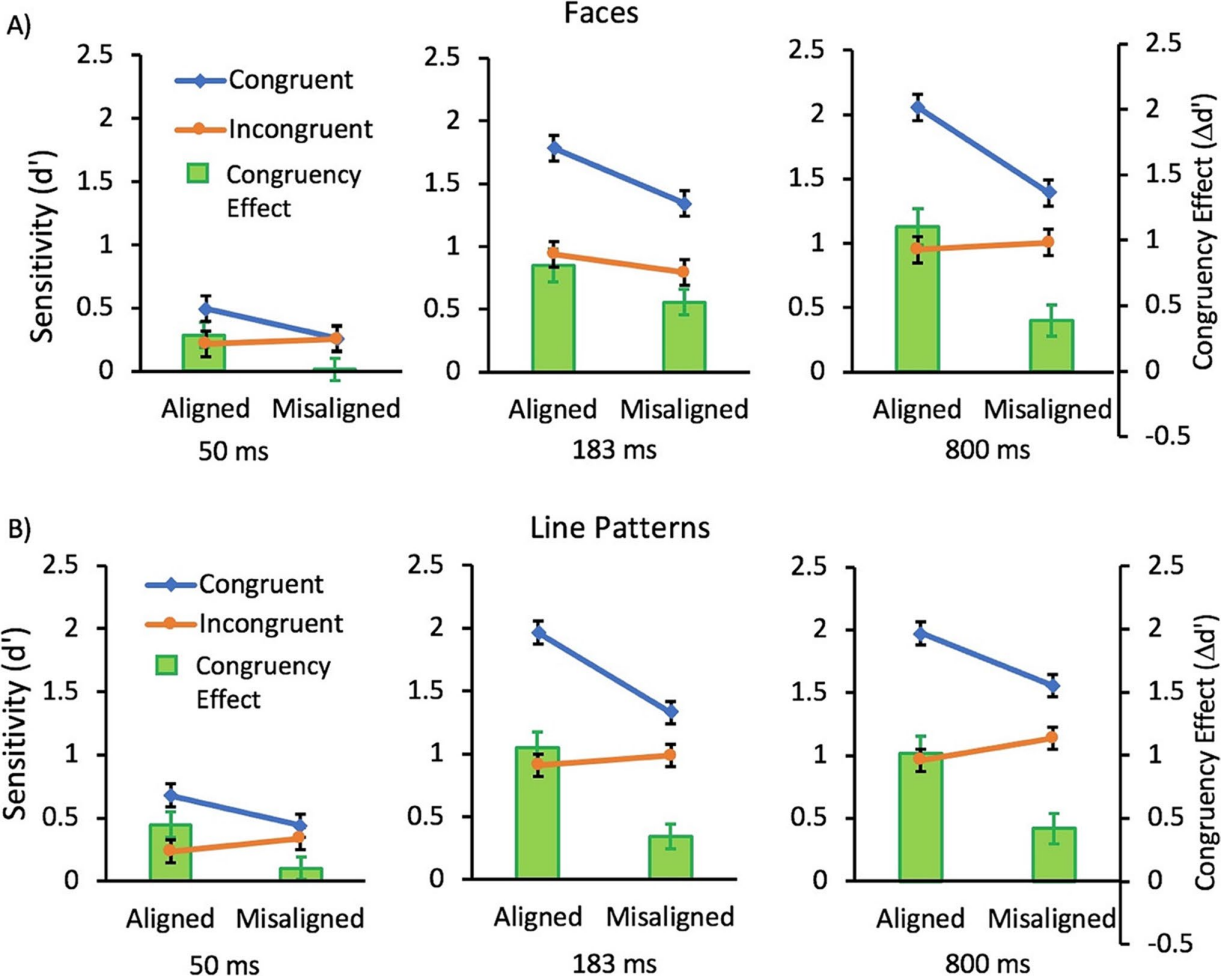
Mean sensitivity (d’) for congruent (blue; diamonds) and incongruent (orange; circles) conditions and the resulting congruency effect (∆d’; green bars) for trials with A) face stimuli and B) line stimuli as a function of alignment for the three presentation durations tested in Experiment 1. Error bars show standard error of the mean

The ANOVA showed main effects of congruency, *F*(1,34) =60.98, *p* < .0001, η_*p*_^2^ =.64, alignment, *F*(1,34) = 23.53, *p* < .0001, η_*p*_^2^ =0.41, and duration, *F*(2,68) = 107.93, *p* < .0001, η_*p*_^2^ =.76. There was also an interaction between congruency and alignment, *F*(1,34) = 26.589, *p* < .0001, η_*p*_^2^ =.44. This interaction was a result of the effect of congruency being larger in the aligned than the misaligned condition. There was also a 2-way interaction between congruency and duration, *F*(2,68) = 22.81, *p* <.0001, η_*p*_^2^ =.40, but not between alignment and duration, *F*(2,68) = 1.70, *p* =.19, η_*p*_^2^ =0.048. The 3-way interaction with duration also failed to reach significance, *F*(2,68) = 3.10, *p* =.052, η_*p*_^2^ =.083 (Fig. 2A). These findings are consistent with previous studies reporting an onset of holistic processing of faces with as little as a 50 ms masked presentation duration (Richler et al., 2011).

#### Response time (RT)

**Fig. 3:**
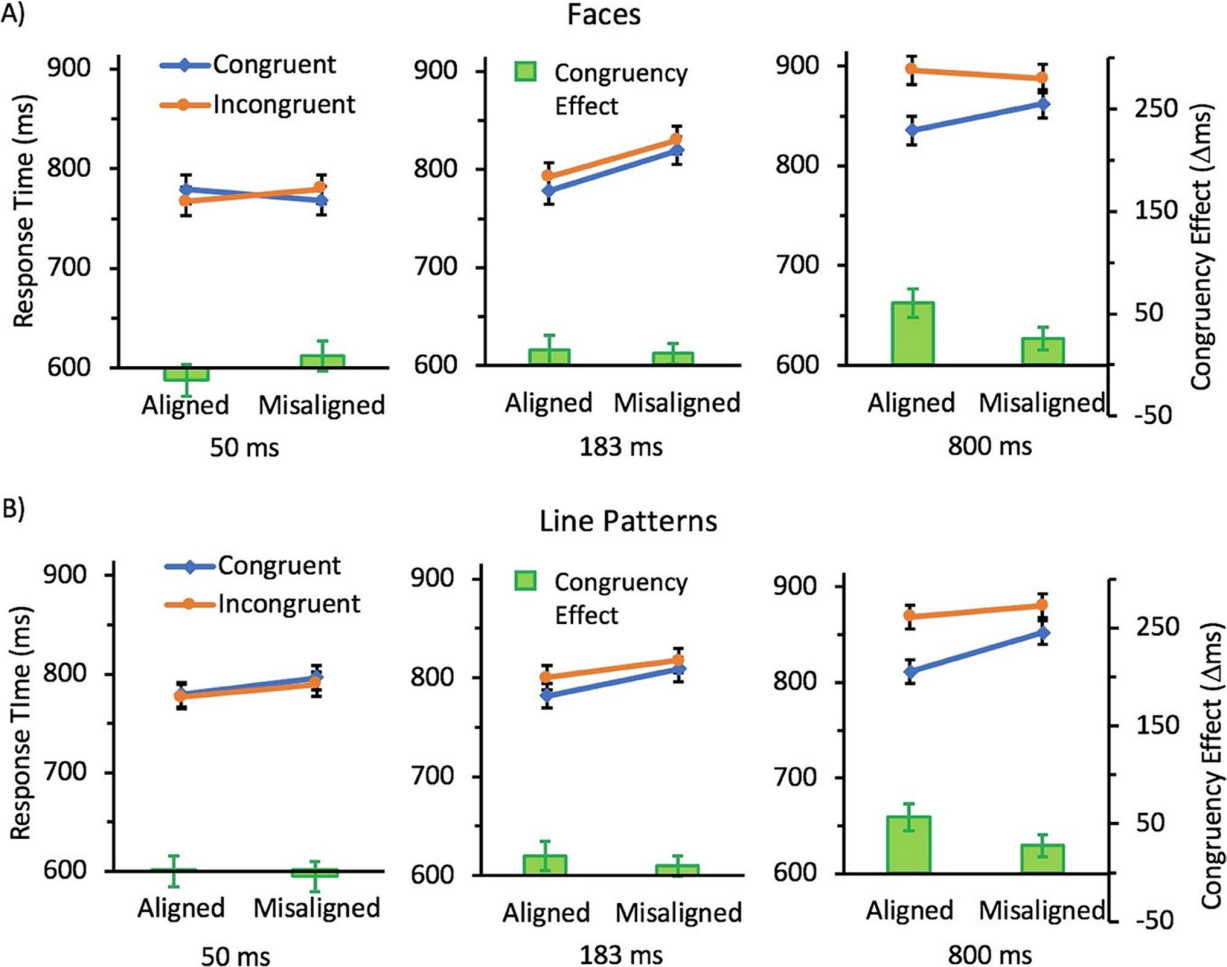
Mean response time (RT) for congruent (blue; diamonds) and incongruent (orange; circles) conditions and the resulting congruency effect (∆RT; green bars) for trials with A) face stimuli and B) line stimuli as a function of alignment for the three presentation durations tested in Experiment 1. Error bars show standard error of the mean

The ANOVA showed main effects of congruency, *F*(1,34) =14.62, *p* = .0005, η_*p*_^2^ =0.81, alignment, *F*(1,334) = 7.17, *p* = .011, η_*p*_^2^ =.17, and duration, *F*(2,68) = 13.28, *p* < .0001, η_*p*_^2^ =.28. There was also an interaction between congruency and duration, *F*(2,68) = 5.64, *p* = .0054, η_*p*_^2^ =.14. Scheffé tests suggest that this interaction was a result of the effect of congruency being non-significant in the 50 ms condition (*p*>.99) and the 183 ms (*p*=.43) conditions, but significant in the 800 ms (*p*=.0001) conditions. The 2-way interaction between alignment and duration was also significant, *F*(2,68) = 4.98, *p* =.0096, η_*p*_^2^ =.13. Scheffé tests suggest that this interaction was a result of the effect of congruency being non-significant in the 50 ms condition (*p*>.99) and the 800 ms (*p*=.59) conditions, but significant in the 183 ms (*p*=.0003) conditions. However, there was no interaction between congruency and alignment, *F*(1,34) = 0.21, *p* =.65, η_*p*_^2^ <.001. There was also no 3-way interaction with duration, *F*(2,68) = 2.08, *p* =.13, η_*p*_^2^ =.06 (see Fig. 3A). While markers of holistic processing equally often emerge in measures of sensitivity or response time, it is not surprising given the generally lower level of performance in the shorter presentation duration conditions that such markers were less robust in the response time data as fewer correct trials were available for the calculation of response time means in these conditions.

There was a trend in the data indicating the possibility of a speed-accuracy trade-off. To address whether this impacted the pattern of results, an additional (not pre-registered) analysis was conducted on the RT data adjusted for accuracy. This was done by calculating inverse efficiency (IE) scores (RT divided by the proportion correct; Townsend & Ashby, 1983). This analysis revealed the same pattern of findings as the RT analysis, that is, main effect of congruency, *F*(1,34) = 61.09, *p* < .0001, η_*p*_^2^ =.64, alignment, *F*(1,34) = 17.018, *p* = .0002, η_*p*_^2^ =.33, and duration, *F*(2,68) = 21.62, *p* < .0001, η_*p*_^2^ =.39, and an interaction between congruency and duration, *F*(2,68) = 13.82, *p* = .0069, η_*p*_^2^ =.29. In addition, this analysis also revealed a significant congruency x alignment interaction, *F*(1,34) = 6.23, *p* = .0181, η_*p*_^2^ =.15, like that found in the sensitivity data. No other effects were significant. Thus, a speed-accuracy trade-off cannot explain this pattern of results.

### Line Pattern Stimuli

#### Sensitivity (d’) data

The ANOVA showed main effects of congruency, *F*(1,34) = 53.46, *p* < .0001, η_*p*_^2^ =.61, alignment, *F*(1,34) = 9.10, *p* =.0048, η_*p*_^2^ =.21, and duration, *F*(2,68) = 143.1, *p* < .0001, η_*p*_^2^ =.81. There was also an interaction between congruency and alignment, *F*(1,34) =43.07, *p* < .0001, η_*p*_^2^ =.56. This interaction was a result of the effect of congruency being larger in the aligned than the misaligned condition. There was also a 2-way interaction of congruency and duration, *F*(2,68) = 7.82, *p* = .0009, η_*p*_^2^ =.19. However, Scheffé tests reveal that the effect of congruency was significant in the 50 ms (*p*=.013), 183 ms (*p*<.0001), and the 800 ms (*p*<.0001) conditions, thus the interaction was likely a result of the smaller effect of congruency in the 50 ms condition. There was no interaction between alignment and duration, *F*(2,68) = 2.92, *p* =.061, η_*p*_^2^ =.08. There was also no 3-way interaction with duration, *F*(2,68) = 2.22, *p* =.12, η_*p*_^2^ =.06 (see Fig. 2B). Thus, like that found for faces, evidence of holistic processing of the line patterns was present with as little as a 50 ms masked presentation duration.

#### Response time (RT)

The ANOVA showed main effects of congruency, *F*(1,34) =9.02, *p* = .0050, η_*p*_^2^ =0.21, alignment, *F*(1,34) = 6.86, *p* =.013, η_*p*_^2^ =.17, and duration, *F*(2,68) = 8.19, *p* =.0006, η_*p*_^2^ =.19. The interactions between congruency and alignment, *F*(1,34) =1.89, *p*=.18, η_*p*_^2^ =.05, and alignment and duration, *F*(1,34) =.35, *p*=.70, η_*p*_^2^ =.01, were non-significant. There was also no 3-way interaction between congruency, alignment, and duration, *F*(2,68) = .55, *p* =.58, η_*p*_^2^ =0.02. However, there was an interaction between congruency and duration, *F*(2,68) = 5.74, *p* = .005, η_*p*_^2^ =.14. This interaction was a result of the effect of congruency being absent in the 50 ms (*p*=.91) and 183 ms (*p*=.39) conditions, but present in the 800 ms (*p*=.0003) condition (see Fig. 3B). As with the response time data from the face conditions, the generally lower level of performance in the shorter presentation duration conditions is likely responsible for the reduced robustness of holistic processing markers in these conditions.

There was again a trend in the data indicating the possibility of a speed-accuracy trade-off. To address whether this impacted the pattern of results, an additional (not pre-registered) analysis was conducted again on the inverse efficiency (IE) scores caculated from the line pattern data (Townsend & Ashby, 1983). This analysis revealed the same pattern of findings as the RT analysis, that is, main effect of congruency, *F*(1,34) = 49.42, *p* < .0001, η_*p*_^2^ =.59, alignment, *F*(1,34) = 8.44, *p* = .0064, η_*p*_^2^ =.20, and duration, *F*(2,68) = 35.99, *p* < .0001, η_*p*_^2^ =.51, and an interaction between congruency and duration, *F*(2,68) = 5.36, *p* = .0069, η_*p*_^2^ =.14. In addition, this analysis also revealed a significant congruency x alignment interaction, *F*(1,34) = 18.62, *p* = .0001, η_*p*_^2^ =.35, like that found in the sensitivity data. No other effects were significant. Thus, a speed-accuracy trade-off cannot explain this pattern of results.

### Comparing Face and Line Pattern Stimuli

Data from the face and line pattern conditions were also compared directly in an a 2 (stimuli; faces, line patterns) x 2 (congruency; congruent, incongruent) x 2 (alignment; aligned, misaligned) x 3 (duration; 50 ms, 183 ms, 800 ms) repeated measures analysis. These analyses were requested during the review process and thus were not included in the pre-registration.

#### Sensitivity (d’) data

This analysis showed no main effect or interactions with category (all ps>.16). There were main effects of congruency, *F*(1,34) = 89.70, *p* < .0001, η_*p*_^2^ =.73, alignment, *F*(1,34) = 34.94, *p* < .0001, η_*p*_^2^ =.51, and duration, *F*(2,68) = 170.73, *p* < .0001, η_*p*_^2^ =.83. There was also an interaction between congruency and alignment, *F*(1,34) =55.15, *p* < .0001, η_*p*_^2^ =.62. This interaction was a result of the effect of congruency being larger in the aligned than the misaligned condition. There was also a 2-way interaction between congruency and duration, *F*(2,68) = 27.66, *p* < .0001, η_*p*_^2^ =.45. However, Scheffé tests reveal that the effect of congruency was significant in the 50 ms (*p*=.002), 183 ms (*p*<.0001), and the 800 ms (*p*<.0001) conditions, thus the interaction was likely a result of the smaller impact of congruency on performance in the 50 ms condition (Δd’=.21) compared to the 183 ms (Δd’=.70) and 800 ms (Δd’=.73) conditions. There was also an interaction between alignment and duration, *F*(2,68) = 3.52, *p* =.035, η_*p*_^2^ =.09, with an effect of alignment in the 183 ms (*p*<.0001) and 800 ms (*p*=.001) conditions, but not the 50 ms condition (*p*=.32). There was also a 3-way interaction between alignment, congruency, and duration, *F*(2,68) = 3.79, *p* =.028, η_*p*_^2^ =.10, with the effect of congruency only present in the aligned (*p*<.0001), but not misaligned (*p*=.68) stimuli in 50 ms duratiom conditons. The effect of congruency was present for both aligned and misalign stimuli in the other duration condtions (all ps<.0001). Thus, there is no evidence that the processing of faces and line patterns differed in terms of the congruency effect, or the interaction between congruency and alignment, as there was no effect of, or interactions with category. Further, these results are consistent with those of the prior analysis, again providing evidence of holistic processing of the stimuli with as little as a 50 ms masked presentation duration.

#### Response time (RT)

This analysis showed no main effect or interactions with category (all ps>.12). There were main effects of congruency, *F*(1,34) =21.18, *p* < .0001, η_*p*_^2^ =.38, alignment, *F*(1,34) = 13.25, *p* < .001, η_*p*_^2^ =.28, and duration, *F*(2,68) = 14.85, *p* < .0001, η_*p*_^2^ =.30. The interactions between congruency and alignment, *F*(1,34) =1.68, *p*=.20, η_*p*_^2^ =.05, and alignment and duration, *F*(1,34) =2.59, *p*=.083, η_*p*_^2^ =.07, were non-significant. There was also no 3-way interaction between congruency, alignment, and duration, *F*(2,68) = 2.30, *p* =.511, η_*p*_^2^ =.06. However, there was an interaction between congruency and duration, *F*(2,68) = 9.48, *p* = .0002, η_*p*_^2^ =.22. This interaction was a result of the effect of congruency being absent in the 50 ms (*p*=.96) and 183 ms (*p*=.23) conditions, but present in the 800 ms (*p*<.0001) condition (see Fig. 3).

## Experiment 2

The results of Experiment 1 provide support for similarities in time-course of the holistic processing of faces and stimuli rich in Gestalt cues. However, while there are times in our everyday lives when we might only get a glimpse of a passing stimulus, such as a face in a crowd, our perceptual processing of those stimuli is rarely curtailed after such a brief exposure, and is usually allowed to continue to operate on representations in iconic memory well beyond 50 ms. It is possible that holistic processing of faces and stimuli rich in Gestalt cues have a similar temporal onset as suggested by the findings of Experiment 1, but they are differentially benefited by additional, post-presentation perceptual processing time. For example, face processing may preferentially benefit from experience-based contributions to holistic processing that may not emerge until later, namely during the post-perceptual processes that were impaired by the presence of a pattern mask in Experiment 1. Experiment 2 examines this possibility by measuring holistic processing of these stimuli when only the presentation duration was constrained, limiting the time participants had to extract information from the physical stimulus, but subsequent processing of this extracted information could continue unimpaired due to the absence of a mask. Thus, the key difference from Experiment 1 was the removal of the pattern mask after the second stimulus.

### Method

#### Participants

Thirty-four undergraduate students (19 males, 15 females, mean age = 23.26, SD = 5.19) from Macquarie University completed the study for class credit. This sample size was chosen to again approximate that in the corresponding condition in Richler et al., 2011. All participants reported normal or corrected to normal vision and gave informed consent before participating.

#### Stimuli & Design

The stimuli and design were the same as that for Experiment 1.

#### Apparatus and Procedure

The apparatus and procedure were the same as that in Experiment 1 except there was no pattern mask presented after the (second) test stimulus. Thus, the screen remained blank with the bracket over the target area until a response (keypress) was made or the trial timed out. The 2 sessions were again completed on separate days, typically a week a part (mean interval = 4.88 days, *S.D*.=2.38).

### Results & Discussion

The data were analysed in the same way as in Experiment 1. Removing trials with extreme RTs (i.e., RTs < 200 ms or >2000 ms) resulted in minimal data loss (1.3%).

#### Face Stimuli

##### Sensitivity (d’)

**Fig. 4.**
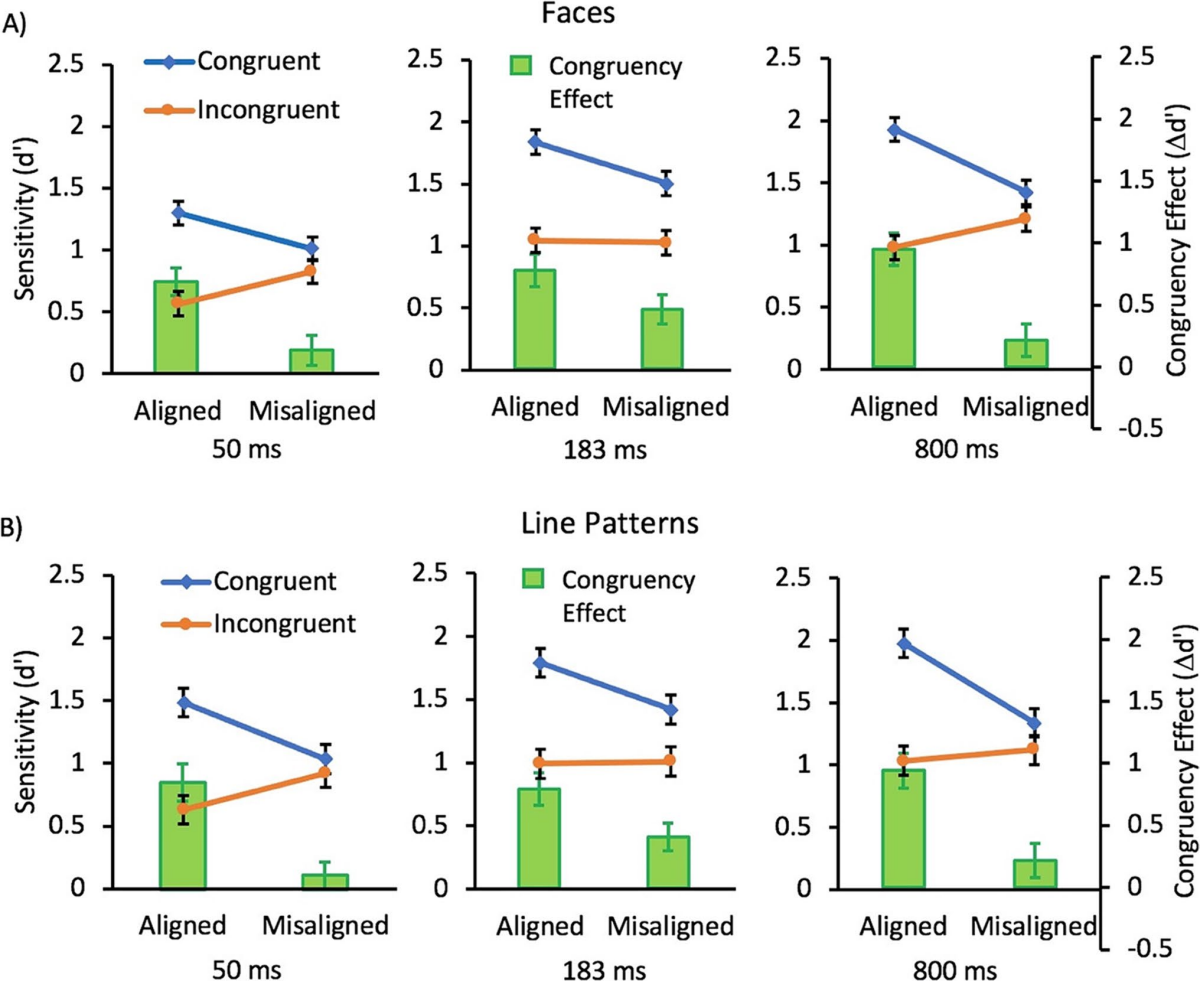
Mean sensitivity (d’) for congruent (blue; diamonds) and incongruent (orange; circles) conditions and the resulting congruency effect (∆d’; green bars) for trials with A) face stimuli and B) line stimuli as a function of alignment for the three presentation durations tested in Experiment 2. Error bars show standard error of the mean

The ANOVA showed main effects of congruency, *F*(1,33) =52.26, *p* < .0001, η_*p*_^2^ =.61, alignment, *F*(1,33) = 8.88, *p* = .0054, η_*p*_^2^ =0.21, and duration, *F*(2,66) = 47.22, *p* < .0001, η_*p*_^2^ =.59. There was also an interaction between congruency and alignment, *F*(1,33) = 35.39, *p* < .0001, η_*p*_^2^ =.52. This interaction was a result of the effect of congruency being larger in the aligned than the misaligned condition. However, there were no 2-way interactions of congruency, *F*(2,66) = 1.27, *p* = .29, η_*p*_^2^ =.04, or alignment, *F*(2,66) = 1.20, *p* =.30, η_*p*_^2^ =0.04, with duration. There was also no 3-way interaction with duration, *F*(2,66) = 2.357, *p* =.10, η_*p*_^2^ =.07. The absence of interaction with duration indicated that there was no significant impact of presentation duration of the stimuli on the size of the congruency or alignment effects for the face stimuli (see Fig. 4A).

##### Response time (RT)

**Fig. 5:**
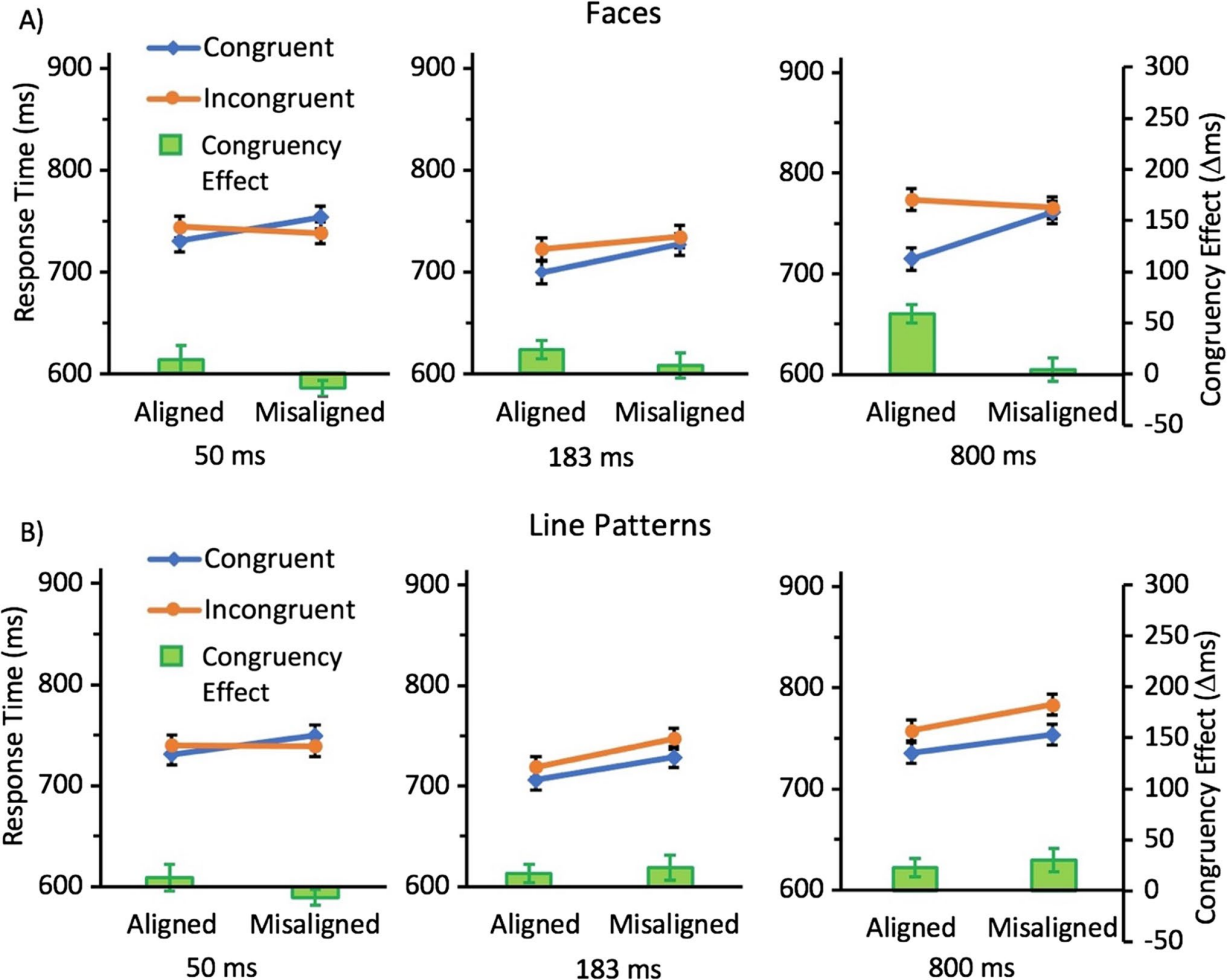
Mean response time (RT) for congruent (blue; diamonds) and incongruent (orange; circles) conditions and the resulting congruency effect (∆RT; green bars) for trials with A) face stimuli and B) line stimuli as a function of alignment for the three presentation durations tested in Experiment 2. Error bars show standard error of the mean

The ANOVA showed main effects of congruency, *F*(1,33) =15.43, *p* = .0004, η_*p*_^2^ =0.32, alignment, *F*(1,33) = 9.79, *p* = .0037, η_*p*_^2^ =.23, and duration, *F*(2,66) = 7.25, *p* = .0014, η_*p*_^2^ =.18. There was also an interaction between congruency and alignment, *F*(1,33) = 12.42, *p* < .0013, η_*p*_^2^ =.27. Scheffé tests suggest that this interaction was a result of the effect of congruency being present in the aligned (p<.0001), but not the misaligned (p=.88) condition. There was also an interaction between congruency and duration, *F*(2,66) = 6.91, *p*=.0019, η_*p*_^2^ =.17. Similar to the findings of Experiment 1, Scheffé tests suggest that this interaction was a result of the effect of congruency being non-significant in the 50 ms condition (*p*=.99), non-significant in the 183 ms (*p*=.056) condition, and significant in the 800 ms (*p*<.0001) conditions. The 2-way interaction between alignment and duration was non-significant, *F*(2,66) = 0.72, *p* =.49, η_*p*_^2^ =.02. There was also no 3-way interaction with duration, *F*(2,66) = 1.66, *p* =.20, η_*p*_^2^ =.05. The absence of three-way interaction indicated that there was no significant impact of presentation duration of the stimuli on the size of the congruency by alignment interaction for the face stimuli in the response time data (see Fig. 5A).

#### Line Pattern Stimuli

##### Sensitivity (d’) data

The ANOVA showed main effects of congruency, *F*(1,33) = 60.45, *p* < .0001, η_*p*_^2^ =.65, alignment, *F*(1,33) = 12.41, *p* =.0013, η_*p*_^2^ =.27, and duration, *F*(2,66) = 23.93, *p* < .0001, η_*p*_^2^ =.42. There was also an interaction between congruency and alignment, *F*(1,33) =47.00, *p* < .0001, η_*p*_^2^ =.59. This interaction was a result of the effect of congruency being larger in the aligned than the misaligned condition. However, there were no 2-way interactions of congruency, *F*(2,66) = .64, *p* = .53, η_*p*_^2^ =.02, or alignment, *F*(2,66) = 1.59, *p* =.21, η_*p*_^2^ =.05, with duration. There was also no 3-way interaction with duration, *F*(2,66) = 1.52, *p* =.23, η_*p*_^2^ =.04. The absence of interaction with duration indicated that there was no significant impact of presentation duration of the stimuli on the size of the congruency or alignment effects for the line pattern stimuli (see Fig. 4B).

##### Response time (RT)

The ANOVA showed main effects of congruency, *F*(1,33) =7.64, *p* = .0092, η_*p*_^2^ =0.19, and alignment, *F*(1,33) = 12.90, *p* =.001, η_*p*_^2^ =.28. The main effect of duration failed to reach significance, *F*(2,66) = 3.09, *p* =.052, η_*p*_^2^ =.09. The interaction between congruency and alignment, *F*(1,33) =.072, *p*=.79, η_*p*_^2^ <.01, and alignment and duration, *F*(1,66) =1.04, *p*=.36, η_*p*_^2^ =.03, were also non-significant. There was also no 3-way interaction between congruency, alignment, and duration, *F*(2,66) = 1.04, *p* =.358, η_*p*_^2^ =0.03. However, there was an interaction between congruency and duration, *F*(2,66) = 3.23, *p* = .046, η_*p*_^2^ =.09. Similar to the findings of Experiment 1, this interaction was a result of the effect of congruency being absent in the 50 ms (*p*=.99) and 183 ms (*p*=.11) conditions, but present in the 800 ms (*p*=.004) condition. The absence of a three-way interaction with duration indicated that there was no significant impact of presentation duration of the stimuli on the size of the congruency by alignment interaction for the line pattern stimuli (see Fig. 5B).

#### Comparing Face and Line Pattern Stimuli

Data from the face and line pattern conditions were also compared directly in an a 2 (stimuli; faces, line patterns) x 2 (congruency; congruent, incongruent) x 2 (alignment; aligned, misaligned) x 3 (duration; 50 ms, 183 ms, 800 ms) repeated measures analysis. These additional analyses were requested during the review process and thus were not included in the pre-registration.

##### Sensitivity (d’) data

This analysis showed no main effect or interactions with category (all ps>.17). There were main effects of congruency, *F*(1,33) = 106.44, *p* < .0001, η_*p*_^2^ =.76, alignment, *F*(1,33) = 24.12, *p* < .0001, η_*p*_^2^ =.42, and duration, *F*(2,66) = 72.88, *p* < .0001, η_*p*_^2^ =.69. There was also an interaction between congruency and alignment, *F*(1,33) =81.36, *p* < .0001, η_*p*_^2^ =.71. This interaction was a result of the effect of congruency being larger in the aligned than the misaligned condition. There was no 2-way interaction between congruency and duration, *F*(2,66) = 1.75, *p* = .18, η_*p*_^2^ =.05. There was also no interaction between alignment and duration, *F*(2,66) = 2.37, *p* =.10, η_*p*_^2^ =.07. However, there was a marginally siginificant 3-way interaction between alignment, duration, and congruency, *F*(2,66) = 3.19, *p* =.048, η_*p*_^2^ =.09, with the effect of congruency only present in the aligned (*p*<.0001), but not misaligned (*p*=.18) stimuli in 50 ms duratiom conditons. The effect of congruency was present for both aligned and misalign stimuli in the other duration condtions (all ps<.03). Thus, like that found for Expeirment 1, there is no evidence that the processing of faces and line patterns differed in terms of the congruency effect, or the interaction between congruency and alignment, as there was no effect of, or interactions with category. Further, these results are consistent with those of the prior analysis of this data, again providing evidence of holistic processing of the stimulu with as little as a 50 ms masked presentation duration.

##### Response time (RT)

This analysis showed no main effect or interactions with category (all ps>.15), except for an interaction between congruency, alignment and category, *F*(1,33) =6.43, *p* = .016, η_*p*_^2^ =0.16, with the effect of misalignment on the congruency effect being greater for faces than for line patterns. Specifcally, an effect of congruency was present for both aligned (*p*=.02) and misaligned (p=.04) line patterns, while such an effect was only present for aligned faces (p<.0001; misaligned faces, *p*=.86). There were main effects of congruency, *F*(1,33) =19.19, *p* = .0001, η_*p*_^2^ =.37, alignment, *F*(1,33) = 21.18, *p* < .0001, η_*p*_^2^ =.39, and duration, *F*(2,66) = 5.92, *p* =.0043, η_*p*_^2^ =.15. The interaction between congruency and alignment, *F*(1,33) =8.00, *p*=.0079, η_*p*_^2^ =.20, also reached significance. However, the interaction between alignment and duration, *F*(1,33) =1.99, *p*=.15, η_*p*_^2^ =.06, was non-significant. There was also no 3-way interaction between congruency, alignment, and duration, *F*(2,66) = .93, *p* =.40, η_*p*_^2^ =.03. However, there was an interaction between congruency and duration, *F*(2,66) = 9.00, *p* = .0004, η_*p*_^2^ =.21. This interaction was a result of the effect of congruency being absent in the 50 ms (*p*=.99), but present in the 183 ms (*p*=.01) and 800 ms (*p*<.0001) conditions.

## General Discussion

The time course of the emergence of holistic processing markers for faces and novel line stimuli rich in Gestalt grouping cues was strikingly similar, with both emerging for masked presentations of as little as 50 ms. Notably, both markers of holistic face processing, that is, the congruency effect and the attenuation of the congruency effect by misalignment, were present with this presentation duration. These findings support accounts suggesting that the overlap in the mechanisms supporting holistic processing for faces and stimuli rich in Gestalt grouping cues has an early temporal locus.

The findings of Experiment 2 further demonstrate that this striking similarity holds even when only the presentation duration, and not post-presentation perceptual processing, is constrained. Together these findings are consistent with an early, and possibly extended, temporal locus for the overlap in the holistic processing of faces and non-face stimuli rich in grouping cues. Alternatively, it is possible that holistic processing is not characterised by an extended process, but rather occurs and is completed early with no additional benefits provided by allowing more extended post-perceptual processing. The growing robustness of the holistic processing markers with additional presentation time is somewhat inconsistent with this possibility. However, further studies are required to better elucidate and compare the full time-course of holistic processing of faces and stimuli rich in Gestalt cues.

These findings also extend those previously reported for faces. Unlike in the Richler et al. (2011) study, we found evidence consistent with a similarly early onset, after as little as a 50 ms presentation, for the congruency effect *and* the congruency by alignment interaction. Evidence of the congruency by alignment interaction for faces was predominantly found in the sensitivity data. However, there was little evidence to suggest that encoding time impacted the congruency by alignment interaction as the interaction between congruency, alignment, and duration failed to reach significance in both Experiments 1 and 2. However, Richler et al. (2011) suggested that the absence of the congruency by alignment interaction for faces presented for 50 ms in their study may have been a result of low performance, with sensitivity scores nearing chance level in some conditions. However, performance in this condition in Experiment 1of the current study was similarly low. Notably, while holistic processing was deemed to be evident in the Richler et al. (2011) study via the presence of the congruency effect only, the reduction of the congruency effect with misalignment is considered by some as the preferred marker of holistic face processing. It has been argued that this marker better taps into more face-specific aspects of holistic processing (Richler & Gauthier, 2014). Thus, here we extend previous findings by demonstrating that the modulation of the congruency effect by alignment for faces can emerge at least as quickly as the congruency effect alone.

Evidence of an early emergence of the congruency effect for faces also came predominantly from the sensitivity data, with additional stimulus encoding time required to see this effect in the response time data. One of the few differences in the findings of Experiment 1 and 2 was the absence of the effect of encoding duration on the congruency effect in the sensitivity data in Experiment 2, when post-perceptual processing was no longer constrained by the presentation of a pattern mask. This was true for both the face and line pattern stimuli. This interaction in Experiment 1 was shown to be a consequence of a reduced effect of congruency on performance in the 50 ms condition. The absence of this effect in Experiment 2 suggests that post-perceptual processing can support holistic processing, thereby reducing the cost of the limited stimulus presentation time in the 50 ms condition. In addition, performance was generally quite low in the 50 ms conditions in Experiment 1, raising the possibility that performance in some conditions, especially the incongruent conditions, may have been limited by a floor effect.

The finding of a similarly rapid emergence of holistic processing markers in performance for both faces and non-face stimuli rich in Gestalt cues provides converging evidence of an overlap in the mechanisms supporting holistic processing and perceptual grouping. There is evidence that the mechanistic overlap in holistic processing of faces and stimuli rich in perceptual grouping cues may be limited to early perceptual processing stages (Curby & Moerel, 2019), not extending to later processing stages where the holistic processing of faces and objects of expertise have been shown to overlap (Curby et al., 2019). Specifically, while concurrently holding non-face objects of expertise in visual working memory reduces holistic processing of face stimuli (Curby & Gauthier, 2014; Gauthier et al., 2003), concurrently holding stimuli rich in Gestalt cues in working memory had no such impact on holistic processing (Curby et al., 2019). Similarly, holding intact, holistically processed, faces, compared to misaligned faces for which holistic processing is attenuated, in working memory has no impact on holistic processing of these stimuli rich in Gestalt cues. Thus, the overlap in processing resources recruited by face and non-face stimuli rich in Gestalt cues appears distinct from the overlap between the processing of face and non-face objects of expertise. The former appears to occur at an earlier, more perceptual stage, potentially during the encoding of the stimuli as it is not at play after encoding when the stimuli are concurrently held in visual working memory.

Together the above findings have led researchers to suggest that there may be dual pathways supporting holistic processing (Curby & Moerel, 2019; Zhao et al., 2015). These potentially include an early stimulus-based pathway (supporting holistic processing of stimuli strong in Gestalt grouping cues) and an experience-based pathway (supporting holistic processing of object of expertise), with both pathways supporting face processing. The similarly early emergence of markers of holistic processing for face and non-face Gestalt stimuli found here is consistent with the overlap in holistic processing of these stimuli occurring at relatively early, perceptual processing stages.

Notably, previous research has provided indirect evidence that holistic processing of objects of expertise also occurs rapidly, with effects of expertise emerging early in processing. Specifically, a previous study comparing the time-course of individuating upright and inverted faces and also objects of expertise and non-expertise, found evidence of a similarly rapid onset of specialised processing for upright faces and non-face objects of expertise, compared to that for inverted faces and objects of non-expertise. Specifically, performance in matching tasks with faces, and with cars amongst car experts, rose above chance after as little as a 48 ms (masked) presentation (Curby & Gauthier, 2009). However, performance on the same task with inverted faces, and with cars amongst car novices, did not reach this same level until the presentation duration was extended to 118 ms. Thus, stimuli that are typically processed more holistically, that is, objects of expertise and upright faces, show a temporal advantage over those processed less holistically. However, the insights into the time course of holistic processing from this previous study are somewhat limited as holistic processing was not measured directly.

One possibility is that, while the overlap between the holistic processing of faces and stimuli rich in Gestalt cues is limited to relatively early processing stages, the overlap between the processing of faces and objects of expertise is more extended, spanning early *and* later processing stages. The delayed onset of holistic processing for inverted, relative to upright, faces could be interpreted as consistent with this possibility (Richler, et a., 2011; Curby & Gauthier, 2009). Specifically, given inverted face stimuli contain the same stimulus-based grouping cues as their upright versions, their delay in processing is more likely a result of our limited experience or expertise with faces in this orientation. Future studies comparing expert and novice groups to examine the time course of the emergence of experience-driven holistic processing of objects of expertise are required to determine whether experience driven holistic processing can emerge just as rapidly as more stimulus-driven holistic processing, such as that supporting holistic processing of these line patterns rich in Gestalt cues.

It is possible that perception- and experience-based contributions to holistic processing may be entwined and thus not easily separated as is suggested by the continued striking similarity in holistic processing of faces and stimuli rich in Gestalt cues even when later, post-perceptual processing is no longer constrained by the presence of a backward pattern mask (Experiment 2). This would be the case if the contributions of such mechanisms are not independent. Consistent with this possibility, Kimchi and Hadad (2002) demonstrated that experience can influence perceptual grouping, or more specifically the speed with which disconnected stimulus features can be grouped into configurations. Specifically, disconnected line fragments in familiar configurations, that is, configurations matching that of an upright letter, were able to prime performance in a subsequent matching task with intact (non-fragmented) letters when they were presented for as little as 40 ms. Consistent with the familiarity of the letter stimuli driving this rapid perception of the configuration (grouping) of these line fragments, this priming effect emerged later for line fragments in an unfamiliar, inverted (180 degree rotated) letter configuration (Kimchi & Hadad, 2002). Lab-based training emphasising stimulus integration has also been shown to facilitate perceptual grouping (Kurylo et al., 2017). Therefore, rather than experience and perceptual grouping making independent contributions to supporting holistic processing of stimuli, experience may serve to bolster perceptual grouping within objects of expertise. Further research should investigate if enhanced perceptual grouping of objects of expertise can, in part, account for the rapid onset of holistic processing markers for objects of expertise.

Intriguingly, it is possible that the richness of grouping cues in face stimuli may contribute to other effects that typically distinguish face processing from the processing of other stimulus types. For example, stimulus elements that can be perceptually grouped into an object have been shown to capture attention (e.g., Kimchi et al., 2007). Moreover, the strength of this grouping impacted the degree to which the resulting object captured attention (Kimchi et al., 2016). Notably, a number of studies have also suggested that face stimuli can also capture attention (e.g., Theeuwes & Van der Stigchel, 2006). Further, there is also evidence that expertise-relevant stimulus features also capture attention (Carrigan et al., 2019). The degree to which the rich perceptual grouping cues present in face stimuli and/or our perceptual expertise with these stimuli contribute to their ability to capture attention is unknown.

It is important to note that the similarly early emergence of holistic processing markers for faces and stimuli rich in Gestalt cues alone does not suggest that the holistic processing of these stimuli recruit overlapping mechanisms. Rather, the finding we report here, in the context of previous research, provides converging evidence of an overlap in the mechanisms supporting holistic processing of faces and those recruited by stimuli rich in perceptual grouping cues. Notably, a differential onset of the timing of holistic processing markers for faces and these stimuli rich in grouping cues would have been inconsistent with this account. Thus, the holistic processing of these two classes of stimuli not only show a similarly rapid onset, but previous work suggests that manipulating the perception (Curby et al., 2016) or presence (Curby et al., 2013; Curby & Entenman, 2016) of grouping cues, impacts holistic processing of faces. Further, consistent with a competition for overlapping mechanisms, concurrently processing face and these non-face stimuli rich in Gestalt cues produced symmetrical interference effects with holistic processing of both stimulus classes showing similar interference effects (Curby & Moerel, 2019).

In conclusion, we replicate previous findings that markers of holistic processing emerge for faces after masked presentations of as little as 50 ms. We extend this finding by showing that holistic processing of novel stimuli rich in perceptual grouping cues is also present under these same temporal processing constraints. The emergence of markers of holistic processing for faces and these novel stimuli under the same temporal limitations is consistent with previous research suggesting that the mechanisms supporting holistic processing of faces and stimuli rich in perceptual grouping cues are at least partially overlapping (Curby et al., 2019; Curby & Moerel, 2019). This similarity in the presence of markers of holistic processing remains even when post-perceptual processing is no longer constrained by the presence of a backward pattern mask. Given the ability of experience to modify both the perception and strength of perceptual grouping, it is an open question as to the degree to which the perceptual properties of face stimuli and/or our extensive experience with this stimuli category, drives their holistic processing. Further, given holistic processing has been linked with expert visual processing more broadly, the answer to this question has considerable implications for the training of perceptual expertise with non-face stimuli. Specifically, this answer would provide insight into the degree to which stimulus properties, relative to our experience, determine the speed and degree of holistic processing that can occur for a given class of stimuli.
